# Genomic epidemiology of *Campylobacter fetus* subsp. *venerealis* from Germany

**DOI:** 10.3389/fvets.2022.1069062

**Published:** 2023-01-19

**Authors:** Mostafa Y. Abdel-Glil, Helmut Hotzel, Herbert Tomaso, Xavier Didelot, Christian Brandt, Christian Seyboldt, Jörg Linde, Stefan Schwarz, Heinrich Neubauer, Hosny El-Adawy

**Affiliations:** ^1^Friedrich-Loeffler-Institut—Federal Research Institute for Animal Health (FLI), Institute of Bacterial Infections and Zoonoses, Jena, Germany; ^2^Institute for Infectious Diseases and Infection Control, Jena University Hospital, Jena, Germany; ^3^Faculty of Veterinary Medicine, Zagazig University, Zagazig, Sharkia, Egypt; ^4^School of Life Sciences and Department of Statistics, University of Warwick, Coventry, United Kingdom; ^5^Institute of Microbiology and Epizootics, Freie Universität Berlin, Berlin, Germany; ^6^Veterinary Centre for Resistance Research (TZR), Freie Universität Berlin, Berlin, Germany; ^7^Faculty of Veterinary Medicine, Kafrelsheikh University, Kafr El-Sheikh, Egypt

**Keywords:** *Campylobacter fetus* subsp. *venerealis*, dairy cattle, bovine genital campylobacteriosis, genomic epidemiology, IS*Cfe1*, WGS, Germany

## Abstract

*Campylobacter fetus* subsp. *venerealis* (*Cfv*) causes bovine genital campylobacteriosis (BGC), a World Organization for Animal Health (WOAH)-listed trade-relevant disease characterized by severe reproductive losses, such as infertility, early embryonic death and abortion in cattle. BGC has significant economic implications that have prompted several countries to adopt stringent eradication and surveillance measures to contain the disease. In Germany, there has been a low incidence of BGC cases over the past 28 years. This study aimed to illustrate the genomic diversity of German *Cfv* strains isolated from different federal states in Germany. This study analyzed 63 *Cfv* strains, collected between 1985 and 2015, by whole-genome sequencing and compared them with genome data of 91 international *Cfv* isolates. The phylogenetic analysis showed that the *Cfv* population is genetically conserved and has geographic clusters. In Germany, one phylogenetic lineage comprising all strains was identified. This German lineage was part of a subclade that probably emerged in the nineteenth century and diversified over time. The results of this study point to a non-recurrent cross-border introduction of *Cfv* in Germany. The BGC control interventions in Germany can be considered successful as no outbreaks were reported since 2015.

## 1. Introduction

Bovine genital campylobacteriosis (BGC), also known as bovine venereal campylobacteriosis (BVC), is a venereal disease caused by *C. fetus* subsp. *venerealis* (*Cfv*), a comma-shaped, microaerophilic, and Gram-negative bacterium (1). *Cfv* is one of three subspecies of the species *C. fetus* which also contains *C. fetus* subsp. *fetus* (*Cff* ) and *C. fetus* subsp. *testudinum* (*Cft*). *Cfv* is characterized by a distinctive tropism to the genital tract of cattle. In early German literature, *Cfv* was referred to as *Vibrio fetus* type 1 (2). This bacterium can be transmitted during natural mating or *via* contaminated semen and equipment, e.g., during artificial insemination. In cows, the infection leads to infertility, early embryonic death and abortion (3). In addition, *Cfv* colonizes the mucous membranes of the male genital tract without causing disease, but causes a lifelong infection (2). Diagnosis of BGC requires accurate differentiation between *Cfv* and *Cff* (4–6). The latter is a gut-associated subspecies that causes sporadic abortions in cattle and can be isolated from aborted fetuses. Although several methods have been developed (4, 6–8), distinction between the two subspecies remains a major hurdle for diagnostic laboratories because of high genetic similarity and common phenotypic traits (5, 9).

BGC may cause significant economic losses to infected herds, especially in endemic regions, and is listed as a notifiable disease by the World Organization for Animal Health (5). The disease is distributed worldwide (1). However, the occurrence of BGC is lower in developed countries than in developing countries, where natural breeding of cattle is widespread (1). The use of artificial insemination in cattle breeding has been associated with a sharp decrease in abortions and is regarded as an effective means of controlling BGC (10).

The overall incidence of BGC in Germany is low (11). In the Eastern German Democratic Republic (GDR), most of Middle Germany (the area of today's federal state of Saxony) was endemic for bovine vibriosis in 1954 (12, 13). However, a decade later the disease was considered almost eradicated (12, 14). No cases of *Cfv* infection were officially reported for the districts of the GDR between 1965 and 1989. Only sporadic outbreaks in bulls caused by *C. fetus* subsp. *fetus* (*Cff*) were registered (15). In the Western Federal Republic of Germany (BRD), BGC was considered endemic in most of the West German states, although in 1958 only few data were available for Lower Saxony, Bavaria, Schleswig-Holstein, Hesse, and North Rhine-Westphalia (16). Since the annually published Animal Epidemic Report of the Federal Republic of Germany contains only few data on the pathogens, a retrospective epidemiological assessment of BGC is difficult.

According to the official figures on BGC in Germany in recent years i.e., after re-unification of both states in 1990, BGC is well-controlled (https://www.tsis.fli.de/). However, the genetic diversity of *Cfv* strains found in the country in the past remained largely unexplored. In this study, we retrospectively examined 63 *Cfv* strains isolated between 1985 and 2015 using whole-genome sequencing. We performed a phylogeographic analysis in the context of available global *Cfv* genomic data to elucidate the origin and the diversity of *Cfv* lineages that had circulated in the country. This study supports the implementation of WGS into the routine monitoring of *Cfv* infections and outbreak investigations.

## 2. Methods

### 2.1. Epidemiological data

The data concerning the occurrence of BGC outbreaks in Germany was obtained from the Animal Disease Information System (https://www.tsis.fli.de/) hosted by the Friedrich-Loeffler-Institut, the German Federal Research Institute for Animal Health. This data was curated from reports submitted by competent veterinary authorities of the federal states to the Federal Ministry of Food and Agriculture following the German Animal Health Act on notifiable animal diseases.

### 2.2. Bacterial strains and whole-genome sequencing

**Figure 1:**
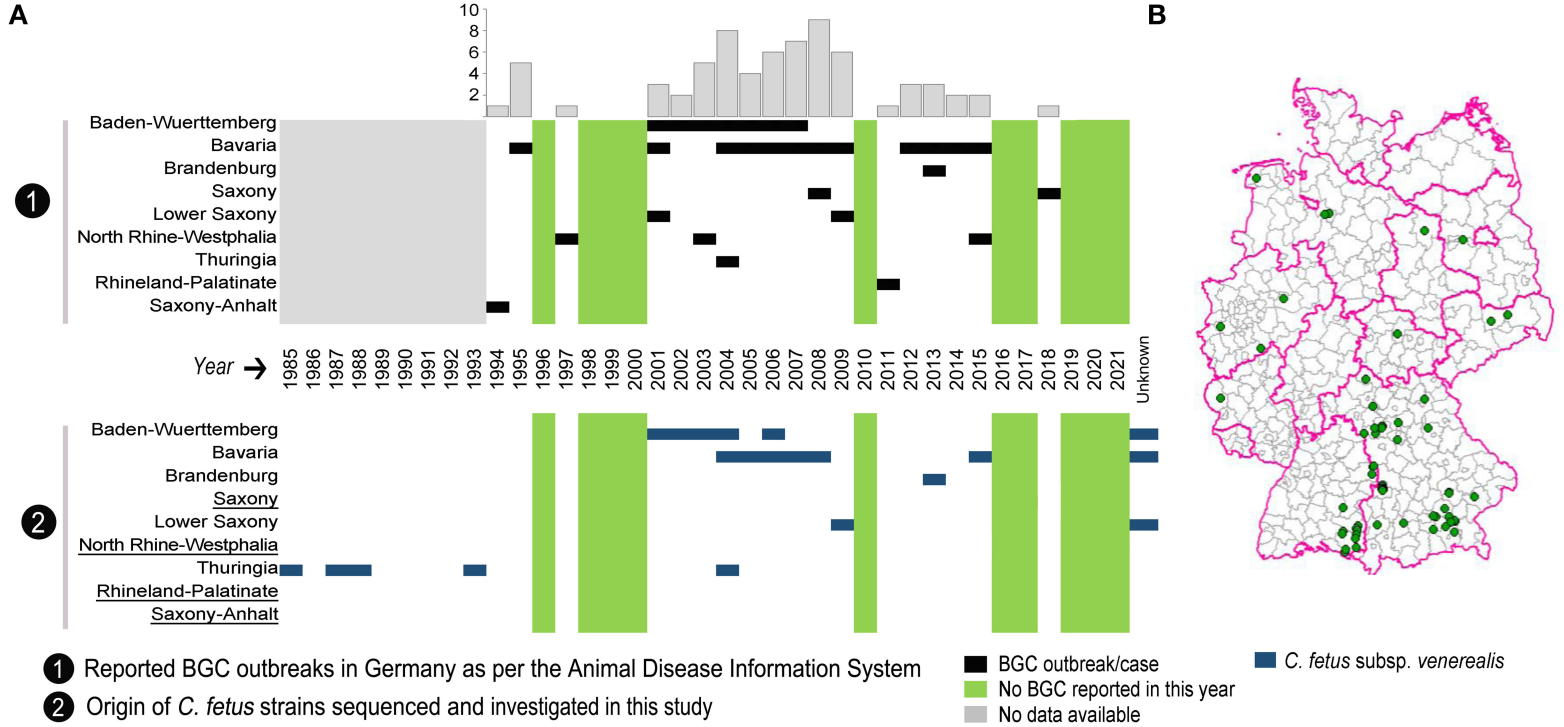
Distribution of reported outbreaks of bovine genital campylobacteriosis (BGC) in Germany over the last 28 years. **(A)** Chronological distribution of the reported BGC outbreaks per German state with the annual cumulative number of outbreaks in the bar chart. The bottom panel shows the temporal distribution of *C. fetus* subsp. *venerealis* strains examined by whole-genome sequencing in each state. Underlined German states indicate that no strains were available for sequencing. **(B)** Map showing the geographical distribution of reported BGC outbreaks across Germany.

Sixty-three *Cfv* strains were provided from the German NRL for Bovine Genital Campylobacteriosis (Figure 1, Supplementary Table S1). Out of 63 strains, 57 and six were collected between 2001 and 2015 and between 1985 and 1993 in Thuringia, respectively. Among them, five *Cfv* in the period 1985 to 1988 were isolated in the GDR (East Germany) and were sent from Thuringian laboratories to the German reference laboratory only after the re-unification of both German states (17).

The genomic DNA was extracted using DNeasy Blood and Tissue kit (QIAGEN^®^, Hilden, Germany) as per the manufacturer's instructions. The DNA was eluted in 200 μl elution buffer. DNA was quantified spectrophotometrically using a Nanodrop^®^ ND-1000 (Fisher Scientific GmbH, Schwerte, Germany). The quality of the DNA was determined using the Qubit dsDNA BR Assay Kit (Invitrogen, Carlsbad CA, USA). The extracted DNA was sequenced using an Illumina MiSeq2000 platform with Nextera XT sequencing library kits to generate 300 bp paired-end reads (Illumina, https://www.illumina.com) and an average sequencing depth of 108-fold (range, 38–226-fold) for all genomes. In addition, six genomes previously sequenced using the Illumina Hi-Seq platform and having an average sequencing depth of 328-fold (range, 88–507) were included (18) (Supplementary Table S1). For all genomes, fastp (v0.23.2) (19) was used to report sequencing metrics followed by taxonomic classification of sequence reads using the Kraken2 database (20). Genome assembly was performed using shovill (v1.0.4) (21) and information about the assembled genomes was collected with seqkit (v2.3.1) (22) (Supplementary Table S3). We deposited the genome sequence data at the National Center for Biotechnology Information (NCBI) under the BioProject accession number PRJNA891116.

For molecular genotyping, all genomes were screened with *cfvCatch* (no version; https://gitlab.com/FLI_Bioinfo/cfvcatch; accessed on 01.2022) (7). *cfvCatch* reports the phylogenetic position of *C. fetus* genomes, as well as 11 nucleotide markers specific for clade 1 genomes (Supplementary Table S1). In addition, it predicts *C. fetus*-specific PCR amplicons, the insertion element IS*Cfe1*, and MLST sequence types (Supplementary Table S1).

### 2.3. Variant calling and phylogenetic analysis

The raw read sequence data obtained from 63 *Cfv* in this study was compared with sequence data from NCBI (Supplementary Table S2). The software snippy (v4.6, https://github.com/tseemann/snippy) was used in default mode to identify sequence variants with the *Cfv* genome 97/608 (accession number CP008810) as a reference. The resulting alignment file was filtered using Gubbins (v2.4.1) (23) and HomoplasyFinder (no version) (24) applying default settings to mask putative recombinant regions and homoplastic SNPs, respectively. Based on a stripped alignment of 1,619 variant sites, a maximum likelihood (ML) tree was built with RAxML-NG (v1.1) (25) using the GTR + Γ nucleotide substitution, Lewis' correction of ascertainment bias and 650 bootstrap replicates (the bootstrapping test estimated applying “majority rules extended” (MRE) was converged after 650 trees). The *Cfv* tree was mid-point rooted based on the phylogenetic structure of the *C. fetus* species (7, 18). The ML tree was visualized using iTOL (v5) (26).

### 2.4. Temporal signal exploration

To estimate the divergence time of *Cfv* lineages, *BactDating* (v1.1; https://github.com/xavierdidelot/BactDating) was applied to the recombination-filtered phylogeny using the additive relaxed clock (ARC) model (27) and 100 million Markov chain Monte Carlo (MCMC) iterations sampled every 10^4^ steps (28). The effective sample sizes (ESS) were computed for the parameters α, μ, and σ >200. The MCMC convergence was verified by visualizing traces of the model parameters. The estimated emergence time of *Cfv* lineages were transposed onto the ML phylogeny produced from SNP analysis.

## 3. Results and discussion

### 3.1. Incidence of BGC outbreaks in Germany

The WOAH defines an outbreak as one or more animals infected with a pathogen, with or without clinical signs, in an epidemiological unit (29). According to this definition, there have been a total of 68 BGC outbreaks in Germany since 1994, as recorded in the Animal Disease Information System reports (https://www.tsis.fli.de/; accessed on 01.03.2022). BGC has been reported in nine federal states, with a range of zero to nine outbreaks per year. The spatial and temporal distribution of reported BGC outbreaks is depicted (Figure 1) and shows that most outbreaks occurred in Southern Germany: Bavaria (*n* = 42) and Baden-Wuerttemberg (*n* = 14), followed by Lower Saxony (*n* = 4), North Rhine-Westphalia (*n* = 3), and Saxony (*n* = 2). One BGC outbreak each was reported in the federal states of Thuringia, Brandenburg, Rhineland-Palatinate, and Saxony-Anhalt, while no reports exist for the other federal states (Figure 1B). From 2001 to 2009, 45 outbreaks, and since 2010, 12 outbreaks were reported.

### 3.2. Use of whole-genome sequencing to reclassify *C. fetus* strains from German BGC

Whole-genome sequencing was used to characterize 63 *C. fetus* strains from German BGC cases. These strains were collected from seven out of nine BGC-affected federal states. Out of them, nine strains had no known date of isolation (three from Baden-Wuerttemberg, one from Bavaria and five from Lower Saxony). No strains were available from North Rhine-Westphalia, Rhineland-Palatinate, Saxony and Saxony-Anhalt (Figure 1, Supplementary Table S1).

All German strains were assigned to clade 1, the bovine-specific clade of *C. fetus* (9, 18), recently proposed as *Cfv* (7). These strains carry IS*Cfe1* and *parA* genes [characteristic markers of *Cfv* subspecies (4)], belong to MLST sequence type (ST) 4 except for one strain of ST75, and show positive alignment to *Cfv*-specific PCR primers with the successful prediction of *Cfv* PCR amplicons (Supplementary Table S1).

### 3.3. A genetically conserved *Cfv* population comprising lineages with geographic characteristics

For a comparative phylogeographic analysis of the 63 German *Cfv* strains, sequences of an additional 91 geographically-diverse strains collected in 11 countries and previously assigned to clade 1 (*Cfv* clade) were downloaded from the NCBI database (Supplementary Tables S1, S2). The mutation rate for the *Cfv* clade was estimated to be 1.47 × 10^−7^ s/s/y (substitutions per site per year; 95% CI = 0.64 × 10^−7^ – 2.2 × 10^−7^), i.e., an average of ~0.3 substitutions in the 2 Mb *Cfv* genome occurs every year. This is 150-fold lower than previous estimates reported for *C. fetus* species (2.9 × 10^−5^ s/s/y), reflecting the conserved genetic diversity possibly due to the clade's restrictive adaptation to cattle (18). Similarly, van der Graaf–van Bloois et al. (30) reported a molecular clock rate of 1.5–3.5 × 10^−2^ substitutions per kb per year for mammalian-associated *C. fetus*, with the *Cfv* genome-cluster having a high dN/dS ratio (0.4) suggesting recent diversification (30). The use of genetic data to estimate evolutionary rates and timescales of pathogens provide valuable insights into their biological processes. These estimates, however, can be influenced by access to informative sequence data and careful model selection (31).

**Figure 2:**
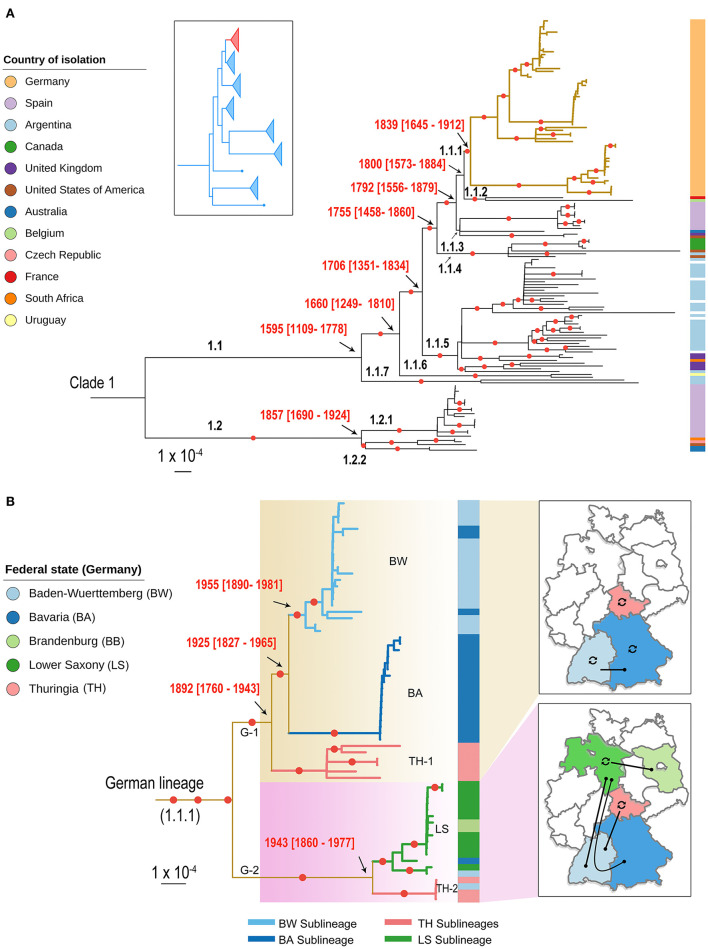
Population structure of *C. fetus* subsp. *venerealis* (*Cfv*) strains. **(A)** A contextual phylogenetic analysis of the 63 German *Cfv* strains (1985–2015) in the light of global data from 11 countries is extrapolated by a midpoint-rooted maximum likelihood (ML) phylogenetic tree of clade 1. The placement of clade 1 (red) in relation to the major clades of the parent *C. fetus* species is shown in the inset box as previously indicated (7). The descending lineages of the *C. fetus* subsp. *venerealis* clade were named hierarchically. The German lineage (lineage 1.1.1) is highlighted with thickened yellow colored branches. **(B)** A pruned phylogeny showing the country-level diversification of the German lineage into two main lineages and five sublineages and their geographic distribution. The five sublineages were labeled according to their major geographic distribution and the branches were colored accordingly. The color bar on the right of the phylogeny in **(A, B)** highlights the geographic origin of the strains, as indicated in the legend. The date of the internal nodes was estimated using BactDating, and the estimated date ranges were superimposed onto the phylogeny. Branches with bootstrap values >90% are marked with a red circle. The branch lengths are in substitutions per core genome site.

The phylogeny of global *Cfv* reveals two main subclades (1.1 and 1.2) and nine descending genetic lineages named hierarchically (Figure 2A). The average number of SNP differences over sequence pairs within the identified lineages was 46 (13–88), between the lineages was 154 (60–245) and between the two main subclades was 215 SNPs. Importantly, some of the identified *Cfv* genetic lineages display distinct geographic features with strains from one country grouped into a single lineage (e.g., Germany and Canada) or in multiple lineages (e.g., Argentina and Spain; Figure 2A). The absence of geographic clustering of some other lineages was mainly confined to regions (and strains) currently underrepresented in our data set.

The so-called South American strains constitute the ancestral population of subclade 1.1 and might be the origin of this subclade. This was extrapolated by first confirming the existence of a significant temporal signal across this clade evidenced by a positive correlation (*R*^2^ = 0.04) between the root-to-tip genetic distance and the sampling times (*p* = 6.51 × 10^−3^; Supplementary Figure 1). The temporal estimates support the phylogenetic branching order from the recombination-corrected SNP analysis. The most recent common ancestor of the South American strains corresponds to the branching of subclade 1.1 into seven lineages which are dated to 1595 (95% CI = 1109–1778), indicating earlier divergence than the other strains (estimated branching from 1660 to 1839; Figure 2A). This was also reflected in the pairwise nucleotide diversity of the South American strains that was higher (52–88 SNP differences) compared to strains from other countries in subclade 1.1 (6–67 SNPs).

### 3.4. An endemic lineage of *Cfv* has been circulating in Germany over the last three decades

Phylogenetic analysis of *Cfv* strains clusters all German strains (1985–2015) into one genetic lineage (1.1.1) within the subclade 1.1 (Figure 2A) with an average pairwise genetic distance of 43 SNPs. Interestingly, this lineage included strains recovered at the time of the GDR (East Germany, TH-1) before re-unification. This suggests that the strains of the GDR and Germany have a common ancestor and that the date of *Cfv* introduction into Germany predates the division of the country after World War II (WWII). We estimated that the most recent common ancestor of the German strains circulated since the nineteenth century (1839; CI: 1645–1912; Figure 2A). The grouping of *Cfv* strains from both German states into one lineage is striking. This lineage might have persisted under the tight physical borders and the different farming and breeding systems present for nearly 45 years in both German states during the Cold War times. For the Thuringian strains isolated in the 1980's, no metadata are available. Thus, it is not clear if they were isolated from endemic cattle as no official records were made. Notification of BCG outbreaks by official state veterinarians can be taken for granted, considering that BCG was a notifiable disease in the GDR. Also, for the Thuringian strain of 1993, no metadata are available and no BGC notification can be identified either. One can speculate that it originated from animal trade as a consequence of the replacement of the population after re-unification which leaves the question unanswered why this sublineage (SL) was found 1985–1988 already in the GDR. These events stay cryptic. Further socio-economic research is also required to clarify why other strains were not introduced into Germany and whether the German lineage was not exported. It is plausible that the spread of *Cfv* across German borders is limited today due to strict eradication programs in Germany and tight international trade controls. In addition, the fact that the host range is limited to cattle restricts the dynamics of the pathogen to the movement of animals or the transport of animal products, such as infected semen, thus promoting control of the spread. A similar observation can be made in Spain (Figure 2A). Those strains (2002–2014) are distributed in two genetically distinct lineages that include only Spanish strains, with a mean pairwise genetic distance of 9 and 13, respectively (Figure 2A).

### 3.5. Geographic distribution of German *Cfv* sublineages

At the country level, the German lineage has diversified into two major lineages [German Lineage 1 (G-1) and G-2] and five sublineages (BW, BA, TH-1, LS, TH-2; Figure 2B). The first lineage (G-1) comprises 44 strains isolated in Southern and Middle Germany. This lineage has a common ancestor dated earlier [1892 (CI: 1790–1943)] than the second lineage [1943 (CI: 1860–1977)] suggesting a later diversification of the second lineage (Figure 2B). The second lineage (*n* = 19 strains) occurs mainly in Northern Germany, but was also occasionally detected in recent years in Southern Germany, Thuringia, and Brandenburg (Figure 2B). Interestingly, the distribution of German *Cfv* lineages is in line with the distribution of German cattle breeds. For example, specialized dairy cattle (Niederungsvieh, lowland cattle) is bred in Northern Germany, while mixed-purpose cattle (Höhenvieh, highland cattle) is bred in Southern Germany (32). This North-South division is further supported by genetic studies of bovine Y-chromosomes. Unfortunately, no data was available on the cattle breed from which *Cfv* was recovered.

The five German sublineages (SL) show the distinct geographic distribution in federal states. Hence, these sublineages were labeled according to the major representative state (Figure 2B). The first SL (BW SL) includes 18 strains from Baden-Wuerttemberg (2001–2006) and three strains from Bavaria (2005, 2006, and 2015; Figure 2B). The clustering of strains from Baden-Wuerttemberg and Bavaria can presumably be explained by the geographic proximity of these two states or a sporadic transmission from Baden-Wuerttemberg to Bavaria. However, the detailed epidemiological information of the three Bavarian strains was unavailable to confirm this.

The second SL (BA SL) was only confined to Bavaria and comprises 17 strains (2004–2008; Figure 2B). Thus, the co-circulation of two SLs (BA SL and BW SL) in Bavaria is likely as Bavaria has the largest cattle population and bull semen production centers nationwide.

The third SL (TH-1 SL) includes six strains labeled and cataloged in Thuringia between 1985 and 1993; five strains date back to the time of the GDR (1985–1987). Interestingly, the GDR strains cluster separately from the other German strains, reflecting the restriction of cattle trade between both states. The fourth SL (TH-2 SL) was also detected in Thuringia, including four identical strains (zero SNPs). Three strains were obtained from prepuce and semen of two bulls in March 2004 in Thuringia and the same strain was obtained from an aborted fetus in Baden-Wuerttemberg one month later, suggesting an epidemiological link.

The fifth SL (LS SL) contains 11 strains from Lower Saxony and two strains isolated from Baden-Wuerttemberg (2004) and Bavaria (2007). It also includes the only two strains isolated in Brandenburg in 2013. The Brandenburg strains are genetically close to Lower Saxony strains (pairwise SNPs, 0–7). The TH-2 SL and LS SL prove that with the replacement of GDR stocks with Western German cattle also *Cfv* strains were introduced to Eastern German federal states, Thuringia and Brandenburg.

## 4. Conclusion

This study has important implications for decision-makers including agricultural and veterinary health authorities. The overall genomic framework presented in this study has the potential to enable prediction of the geographic origin of *Cfv* strains at inter- and intra-national levels. This conclusion is supported by the findings that *Cfv* lineages share common geographic features and have a very slow mutation rate over time. However, for a broader use, this platform must be updated with strains from regions that are not covered or are poorly sampled. For example, except for Spain and the United Kingdom, there was very limited genomic data on *Cfv* available from all other European countries, including Germany's neighbors France, the Czech Republic and Belgium (only one genome was available for each country). Importantly, all German *Cfv* form a phylogenetic lineage, suggesting endemicity of a single lineage with the last outbreak occurring in 2015. The fact that this endemic lineage has not been detected since 2015 may indicate its eradication due to the stringent measures taken to control BGC in Germany. However, continuous surveillance programs should be in place and should not be limited to bull stations but should include suckler cow husbandry with breeding bull as well. Furthermore, these control measures need to be continuously evaluated in the light of our results to enable traceability of new outbreak strains. The results of this study further support the benefits of integrating WGS into routine monitoring of BGC outbreaks.

## Data availability statement

The data presented in the study are deposited in the National Center for Biotechnology Information (NCBI) repository, BioProject accession number PRJNA891116.

## Author contributions

MA-G, HH, and HT conceived the study. MA-G conducted the analysis with input from XD. HN comprehensively researched and summarized the literature on cattle breeds and BGC in Germany. MA-G and HN wrote the manuscript. HH obtained and characterized all *C. fetus* strains in the study. HT, CB, CS, JL, SS, and HE-A critically revised and commented on the manuscript. All authors approved the publication of the manuscript.

## Supplementary material

Supplementary Figure 1 Temporal signal in the clade 1 *C. fetus* subsp. *venerealis* genomes. Linear regression (right panel) of the root-to-tip distance vs. the sampling date for strains with dotted lines denote the 95% CI. The left panel depict a mid-point phylogeny of the clade 1 *C. fetus* subsp. *venerealis* genomes.

Supplementary Table S1 Summary of *Campylobacter fetus* subsp. *venerealis* strains isolated from the federal states of Germany. The spatial and temporal distribution of these strains is depicted in Figure 1.

Supplementary Table S2 Summary of 91 *Campylobacter fetus* genomes collected in 11 countries and used for the contextual phylogenetic analysis of the German *C. fetus* subsp. *venerealis* strains.

Supplementary Table S3 Raw read and genome assembly statistics for the data set used in this study.
