# Supplementary figures and images for: Genomic epidemiology of Campylobacter fetus subsp. venerealis from Germany
Source: Front Vet Sci. 2023 Jan 19;9:1069062. doi: 10.3389/fvets.2022.1069062 (PMC9893283; doi:10.3389/fvets.2022.1069062)

Rate=1.98e-01,MRCA=1242.97,R2=0.04,p=6.51e-03

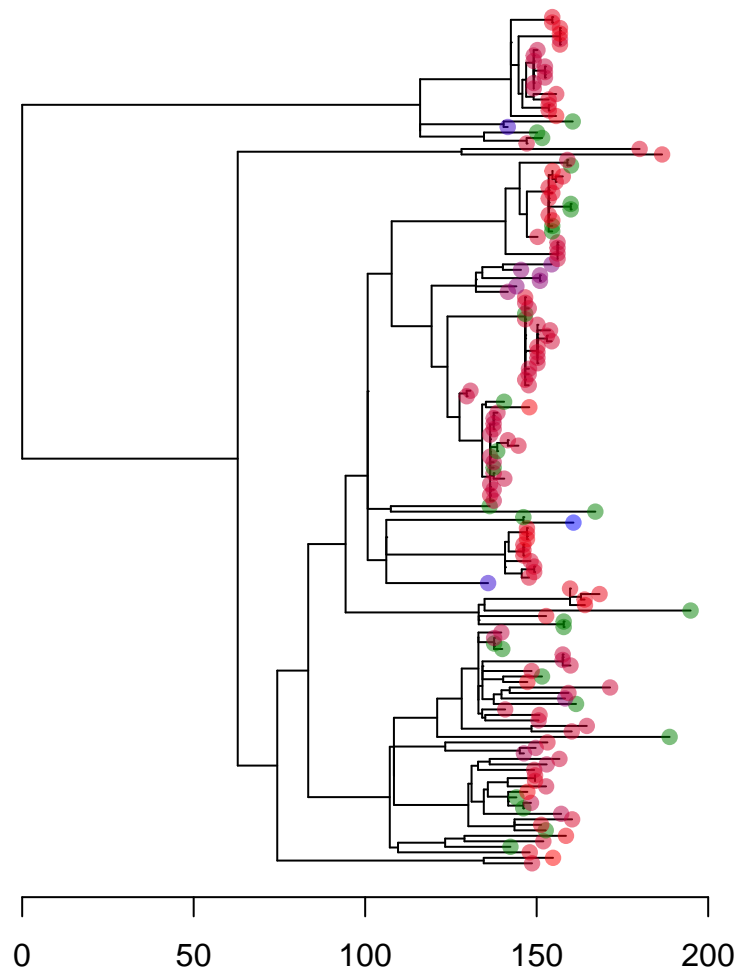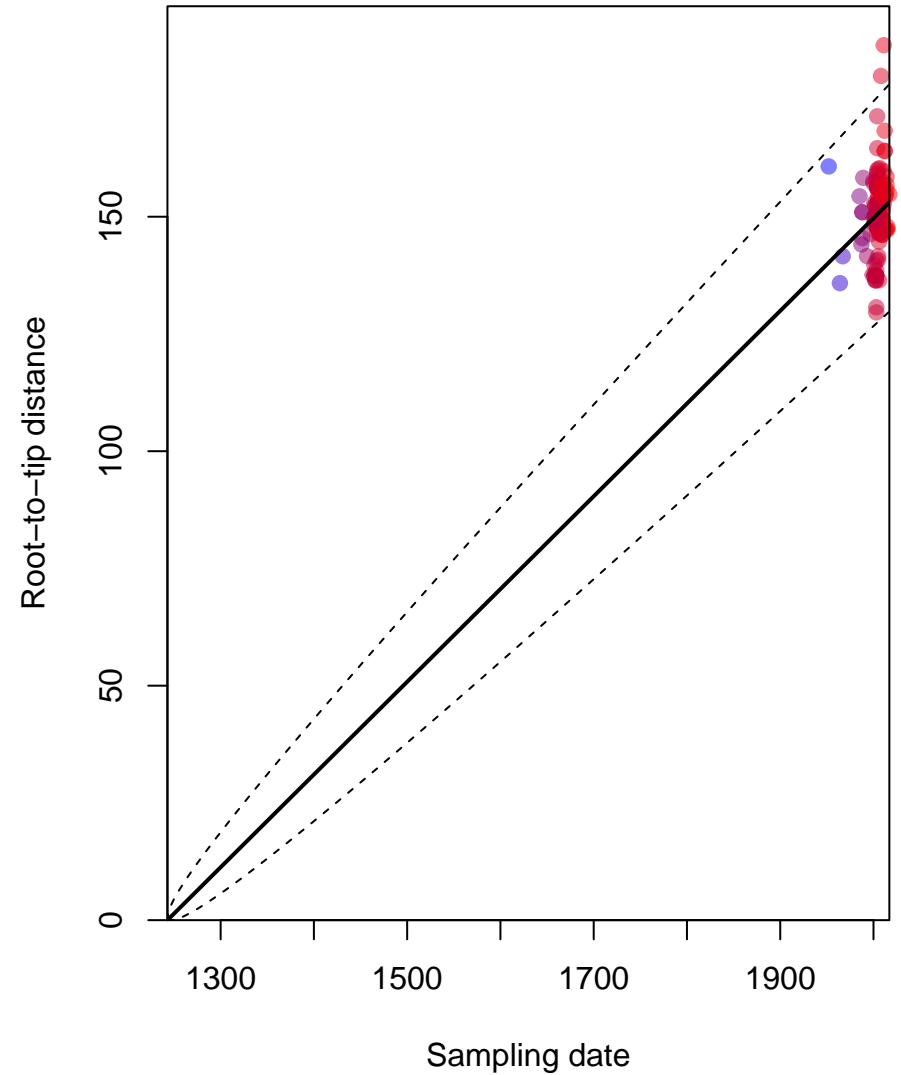

Supplement: Supplementary Figure 1 — Temporal signal in the clade 1 C. fetus subsp. venerealis genomes. Linear regression (right panel) of the root-to-tip distance vs. the sampling date for strains with dotted lines denote the 95% CI. The left panel depict a mid-point phylogeny of the clade 1 C. fetus subsp. venerealis genomes. [file Image_1.PDF]
